# A Comparison of the Clinical Characteristics of Short-, Mid-, and Long-Term Mortality in Patients Attended by the Emergency Medical Services: An Observational Study

**DOI:** 10.3390/diagnostics14121292

**Published:** 2024-06-19

**Authors:** Rodrigo Enriquez de Salamanca Gambara, Ancor Sanz-García, Carlos del Pozo Vegas, Raúl López-Izquierdo, Irene Sánchez Soberón, Juan F. Delgado Benito, Raquel Martínez Diaz, Cristina Mazas Pérez-Oleaga, Nohora Milena Martínez López, Irma Domínguez Azpíroz, Francisco Martín-Rodríguez

**Affiliations:** 1 Emergency Department, Hospital Universitario Rio Hortega, 47012 Valladolid, Spain; renriquezd@saludcastillayleon.es (R.E.d.S.G.); rlopeziz@saludcastillayleon.es (R.L.-I.); 2 Faculty of Health Sciences, Universidad de Castilla la Mancha, 45600 Talavera de la Reina, Spain; 3 Faculty of Medicine, Universidad de Valladolid, 47011 Valladolid, Spain; cpozove@saludcastillayleon.es (C.d.P.V.); francisco.martin.rodriguez@uva.es (F.M.-R.); 4 Emergency Department, Hospital Clínico Universitario, 47003 Valladolid, Spain; 5 CIBER of Respiratory Diseases (CIBERES), Institute of Health Carlos III, 28029 Madrid, Spain; 6 Advanced Life Support, Emergency Medical Services (SACYL), 47007 Valladolid, Spain; msanchezso@saludcastillayleon.es (I.S.S.); jdelgado@saludcastillayleon.es (J.F.D.B.); 7 Department of Project Management, Universidad Europea del Atlántico, 39011 Santander, Spain; raquel.martinez@uneatlantico.es (R.M.D.); cristina.mazas@uneatlantico.es (C.M.P.-O.); nohora.martinez@unini.edu.mx (N.M.M.L.); irma.dominguez@uneatlantico.es (I.D.A.); 8 Department of Project Management, Universidad Internacional Iberoamericana, Campeche 24560, Mexico; 9 Department of Project Management, Universidad de La Romana, La Romana 22000, Dominican Republic; 10 Department of Project Management, Universidad Internacional Iberoamericana, Arecibo 00613, Puerto Rico; 11 Department of Project Management, Universidade Internacional do Cuanza, Cuito EN250, Angola; 12 Fundación Universitaria Internacional de Colombia, Bogotá 111321, Colombia

**Keywords:** predictive models, emergency medical services, long-term mortality

## Abstract

Aim: The development of predictive models for patients treated by emergency medical services (EMS) is on the rise in the emergency field. However, how these models evolve over time has not been studied. The objective of the present work is to compare the characteristics of patients who present mortality in the short, medium and long term, and to derive and validate a predictive model for each mortality time. Methods: A prospective multicenter study was conducted, which included adult patients with unselected acute illness who were treated by EMS. The primary outcome was noncumulative mortality from all causes by time windows including 30-day mortality, 31- to 180-day mortality, and 181- to 365-day mortality. Prehospital predictors included demographic variables, standard vital signs, prehospital laboratory tests, and comorbidities. Results: A total of 4830 patients were enrolled. The noncumulative mortalities at 30, 180, and 365 days were 10.8%, 6.6%, and 3.5%, respectively. The best predictive value was shown for 30-day mortality (AUC = 0.930; 95% CI: 0.919–0.940), followed by 180-day (AUC = 0.852; 95% CI: 0.832–0.871) and 365-day (AUC = 0.806; 95% CI: 0.778–0.833) mortality. Discussion: Rapid characterization of patients at risk of short-, medium-, or long-term mortality could help EMS to improve the treatment of patients suffering from acute illnesses.

## 1. Introduction

Emergency medical services (EMS) constitute the gateway to health care systems for patients with acute conditions. EMS are confronted every day with acute illnesses demanding precise responses in short intervals of time. On arrival at the scene, the EMS must rapidly assess the clinical characteristics of the patient in order to determine the severity of their condition, perform appropriate and timely therapeutic support, and, if necessary, transfer the patient to a referral hospital center [1,2].

To standardize the clinical presentations in prehospital critical care, novel scoring systems have been proposed to evaluate and predict the risk of early clinical worsening [3]. Currently, precision medicine has made great strides in improving prehospital care and emergency departments (EDs), providing bedside scores composed of various clinical, physiological, comorbidity-related, and/or analytical variables as invaluable support in the decision-making process [4].

However, acute illnesses can endanger survival not only at the first stages, but sometimes even days, months, or years later, at which clinical worsening could supervene. Patients admitted to intensive care units (ICUs) exhibit higher mortality rates than the general population, even several years after hospital discharge [5,6]. Several investigations show that this excess mortality is present in patients treated by both EMS and ED after the onset of severe acute disease [7,8]. On the other hand, aging appears to be a key contributor to this mortality; however, evidence suggests that other factors, such as the degree of senescence or the previous functional reserve, play a critical role in excess mortality [5]. Indeed, younger patients admitted to the ICU due to respiratory disease and with a significant comorbidity burden have significantly elevated long-term mortality rates compared to elderly patients without preexisting conditions and infectious diseases [9].

Prehospital clinical characteristics may be useful for categorizing short-term versus long-term mortality. Current risk prognostic models assume a linear relationship between risk factors and clinical outcomes; in contrast, day-to-day patients can present heightened complexity involving a multiplicity of interrelated conditions [10]. In this sense, machine learning or algorithms developed based on artificial intelligence allow the analysis of massive amounts of data and the subsequent development of predictive models. These real-time tools, delivered electronically, will help us to gain a better understanding of the complexity of prehospital critical care landscapes, support clinical decision-making, increase the accuracy and timeliness of diagnosis, and provide prognostic predictions [11,12].

The goal of the present study is to compare the characteristics (all the prehospital predictors, including demographic variables; standard vital signs; prehospital laboratory tests; and comorbidities) of patients presenting short-, mid-, or long-term mortality, and to derive and validate three risk models to determine the aforementioned mortality outcomes.

## 2. Materials and Methods

### 2.1. Study Design

A prospective, multicenter, ambulance-based study was conducted on adult patients (>18 years) with unselected acute disease who were evaluated and managed by EMS and transferred to the ED.

Data were extracted from two consecutive studies conducted under identical design standards: “Prehospital identification of prognostic biomarkers in time-dependent diseases -HITS study-” (ISRCTN48326533) and “Identification of biomarkers of clinical-risk deterioration in prehospital care—preBIO study-” (ISRCTN49321933), which followed the STrengthening the Reporting of OBservational studies in Epidemiology (STROBE) statement (Supplementary Materials, p3) and were approved by the institutional review board of the Public Health Service (reference: PI-049-19/PI-GR-19-1258).

### 2.2. Study Setting

From 8 October 2019 to 31 January 2022, forty-eight basic life support (BLS), five advanced life support (ALS), and four hospitals (one minor general district hospital and three university tertiary hospitals) in the Spanish provinces of Salamanca, Segovia, and Valladolid (Spain) participated in the study. The Public Health System (SACYL) managed and coordinated all medical services.

In an inbound call center (1-1-2 phone number), a teleoperator geo-locates and profiles the emergency. Next, the call is transferred to a medical dispatcher (a physician or registered nurse), who, via a guided interview, dispatches the most appropriate emergency ambulance team to the incident, i.e., an ALS—composed of two emergency medical technicians (EMTs), an emergency registered nurse (ERN), and a physician—or a BLS, formed by two EMTs. All EMS providers adhered to international guidelines for BLS and ALS.

### 2.3. Population

Consecutive adult patients (>18 years) with acute disease who were transported to the ED were enrolled uninterruptedly. For inclusion in the study, every patient had to be examined by the ALS physician, and based on the objective and structured clinical evaluation and the complementary tests available bedside, the physician decided the need for transfer to the ED and the ambulance type (ALS or BLS).

Cases involving minors; unobserved and unrecovered cardiac arrest; ongoing psychiatric disorders; documented end-stage disease; pregnant women (evident or probable); refusal of transfer; failed prehospital blood test; lack of informed consent; and inability to complete follow-up (365 days) were excluded.

### 2.4. Outcome

The primary outcome was noncumulative mortality (all-cause and in- and out-hospital) in the following time windows: short-term mortality (30 days), mid-term mortality (31 to 180 days), and long-term mortality (181 to 365 days). All non-survivor patients were reassessed by the PI.

Secondary outcomes included prehospital and hospital ALS (noninvasive and invasive mechanical ventilation and/or vasoactive agents) and ICU admission.

### 2.5. Data Collection

ALS providers collected the epidemiological variables (sex at birth, age, attention area, nursing home residence, and type of ambulance for transfer) during the first contact with the patient. Next, the ERN measured the complete standardized set of vital signs (respiratory rate, oxygen saturation, blood pressure, heart rate, temperature, and Glasgow coma scale) and proceeded to perform the prehospital blood test (venous blood gases, ions, hemoglobin, renal panel, lactate, and glucose), with simple fresh venous blood drawn at the same time as the venous line was cannulated, as part of a workflow. ALS physicians gathered data concerning electrocardiographic rhythm and ST segment disturbance, as well as prehospital ALS or special follow-up (advanced airway management and/or use of vasoactive agents) and suspected prehospital diagnoses. A LifePAK^®^ 15 monitor–defibrillator (Physio-Control, Inc., Redmond, WA, USA) was used to determine blood pressure, oxygen saturation, heart rate, temperature, and electrocardiographic rhythm. Biomarkers were assessed with Siemens 10,736,515 EPOC BGEM BUN Test Cards using the epoc^®^ POC instrument (Siemens Healthcare GmbH, Erlangen, Germany) following the manufacturer’s instructions.

Finally, a research associate from every hospital, via electronic medical record check after a one-year follow-up of prehospital support, collected the seventeen comorbidity categories to compute the age-adjusted Charlson comorbidity index (aCCI), hospital admissions, ICU admission, advanced airway management and/or use of vasoactive agents, and noncumulative mortality (all-cause and in- and out-hospital).

### 2.6. Statistical Analysis

Descriptive results and the associations between the outcomes and the analyzed variables were assessed by Student’s *t*-test, the Mann‒Whitney U test, or the chi-squared test, when appropriate. Absolute values and percentages were used for categorical variables, and median interquartile ranges (IQR) were used for continuous variables because they did not follow a normal distribution. To determine the variables associated with the outcome, the following process was performed for each noncumulative mortality. (i) A univariate comparison was used to select variables with *p* < 0.001 criterion. (ii) The selected variables were included in a multivariate logistic regression with forward and backward stepwise variable selection. Note that continuous variables included in the model were not categorized. The results from the logistic regression were evaluated using model metrics (Akaike’s Information Criteria (AIC) and Bayesian information criteria (BIC)) and the area under the curve of the receiver operating characteristic curve (AUC). Moreover, the models were internally validated by bootstrapping (1000 iterations), and the Nagelkerke R2 index and the Somers’ Dxy index were reported. Data were collected and registered in a database generated with IBM SPSS Statistics for Apple version 20.0 software (IBM Corp, Armonk, NY, USA). The caseload entry system was tested in order to delete unclear or ambiguous items and to verify the adequacy of the data-gathering system. Missing values were completely random; therefore, a listwise deletion method was used since it does not induce biased means, variances, or regression weight modification (note that three patients were removed from the final predictive model procedure due to missing values). The sample size needed for the present study was *n* = 185, based on the following considerations: a statistical power (1 - β) of 80%, a significance level (α) of *p* = 0.05, a proportion of the sample in the case group (q1) = 0.1, and an estimated odds ratio of 2.

All calculations and analyses were performed using our own codes, as well as R packages and base functions in R, version 4.2.2 (http://www.R-project.org, the R Foundation for Statistical Computing, Vienna, Austria accessed on 18 June 2023).

## 3. Results

A total of 4830 patients were included in the final analysis cohort (see Supplementary Materials, Figure S1), with noncumulative mortalities at 30, 180, and 365 days of 10.8% (523 cases), 6.6% (321 cases), and 3.5% (170 cases), respectively. The median age was 64 years in survivors and 79 years in non-survivors. In a total of 1615 cases (42.3%), the survivors were female; this was also the case for 200 (38.2%), 127 (39.6%), and 60 (35.3%) of the noncumulative mortalities at 30, 180, and 365 days, respectively.

Two-thirds of the patients underwent ALS, climbing to 79.7% in the 30-day non-survivors. Cases with a major comorbidity burden and nursing home origin had significantly elevated mortality rates in all analyzed periods (see Table 1). Table 2 reports the numerical distribution of mortality according to the age-adjusted Charlson comorbidity index. On-scene vital signs, baseline cardiac rhythms, and prehospital blood tests are listed in Table 1.

Short-term mortality cases presented a superior incidence of ALS interventions on-scene, with 12.4% of noninvasive mechanical ventilation, 30.6% of invasive mechanical ventilation, and 16.3% of vasoactive agents, with a marked incidence of acute life-threatening illness, especially sepsis (19.5%), stroke (18.5%), and cardiac arrest (10.3%). As the time window lengthens, the causes of mortality change, highlighting exacerbations of preexisting comorbidities, e.g., heart failure, chronic obstructive pulmonary disease/dyspnea, or syncope. Hospital inpatient admittance rates, ICU admissions, and hospital ALS interventions were most intense in the short-term mortality cluster, showing a linear decrease in the rest of the mortality groups and the lowest incidence in survivors (Table 3).

Table 4 summarizes the predictive models of each mortality. Some of the final selected variables were repeated for all three models (age, partial pressure of carbon dioxide, hemoglobin, and aCCI), but some were exclusive to one outcome—for 30-day mortality, heart rate, ocular Glasgow coma scale, calcium, chlorine, and creatinine were exclusive; for 180-day mortality, nursing home origin, oxygen saturation, tachyarrhythmia, and pH were exclusive; and for 365-day mortality, no variables were exclusive. It is important to highlight that the oxygen saturation/fraction of inspired oxygen ratio was associated with both 30-day and 365-day mortality; for the last case, except for hemoglobin, no analytical parameters were selected. The metrics used to evaluate the models showed that they improved as we considered fewer variables (as better models occurred when increasing mortality time), with results of 1779 and 1901, 1785 and 1893, and 1208 and 1246, respectively, for the AIC and BIC of 30-, 180-, and 365-day mortality. Supplementary Tables S1, S2, and S3 show the univariate analyses of 30-, 180-, and 365-day mortality, respectively.

**Figure 1:**
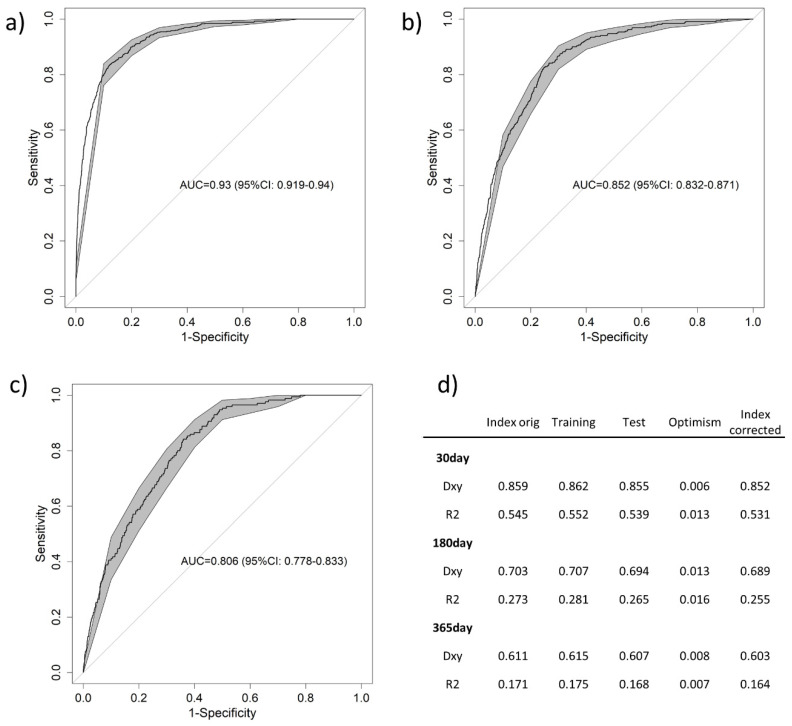
Area under the curve of the receiver operating characteristic curve for (**a**) 30-day, (**b**) 180-day, and (**c**) 365-day noncumulative mortality. Panel (**d**) shows the internal validation results. Abbreviations: R2: the Nagelkerke R^2^ index; Dxy: Somers’ Dxy index.

Finally, the models’ predictive values were assessed (Figure 1), and the best predictive value was shown for 30-day mortality (Figure 1a) (AUC = 0.930, 95% CI: 0.919–0.940), followed by 180-day mortality (Figure 1b) (AUC = 0.852, 95% CI: 0.832–0.871) and 365-day mortality (Figure 1c) (AUC = 0.806, 95% CI: 0.778–0.833). These results were confirmed by the internal validation parameters (Figure 1d).

## 4. Discussion

To our knowledge, the present prospective, multicenter, ambulance-based study, conducted in adults with unselected acute disease who were evaluated and managed by EMS and transferred to the ED, is unique because it analyzed on-scene point of care testing (POCT) and demographic, epidemiological, physiological, electrocardiographic, and comorbid characteristics to detect short-, mid-, and long-term mortality.

Our short-term mortality (30 days) was higher (10.8%) than that in a large study carried out in the Danish National Health System involving 219,323 patients transported by ambulance, with a cumulative 30-day mortality of 7.2% [13]. Nonetheless, it was significantly lower than that obtained in another study in Finland on patients transported by Helicopter Emergency Medical Service, which showed a 30-day mortality prevalence of 27% [7]. Such differences could result from the assumption that the Danish study analyzed all the patients transferred, and the Finnish study included patients referred with high priority by helicopter, whereas our study involved selected patients previously evaluated by physician or nurse dispatchers (1-1-2 emergency coordination center) and tagged as high-priority patients by the on-scene ALS physician. Accordingly, the pretest probability of our study appears to range in the middle of the two populations under analysis, suggesting a relationship with the mortality observed in each study.

The cluster of mid-term mortality (31 to 180 days) and long-term mortality (181 to 365 days) has been under-studied in the scientific literature but, in terms of mortality rate, was very comparable to the cumulative 1-year mortality of surviving patients admitted to an ICU [14], or to the 31–365-day mortality cluster in the aforementioned Finnish study [7]. The observed excess mortality in patients following acute life-threatening illness and after ICU admission could result from post-intensive care syndrome (PICS), characterized by a combination of physical, cognitive, and mental symptoms involving a post-discharge deterioration in quality of life and correlated with poorer long-term outcomes [15]. A comparable pathophysiological mechanism may be observed in patients managed by EMS in critical condition who require prehospital critical care [9,16,17].

The short-term mortality model showed excellent prognostic performance (AUC = 0.93), outperforming other scoring systems used to detect early mortality and comprising variables such as age, comorbidities, vital signs, and lactate [13,18]. Lactate has been extensively reported as a quick biomarker of metabolic stress and tissue hypoxia, and has been demonstrated to be a strong predictor of short- and long-term mortality in several clinical circumstances, including prehospital care [19]. However, other biomarkers included in the model remain less well known [calcium, chloride, hemoglobin, creatinine, and blood urea nitrogen]. Hypocalcemia, decreased hemoglobin, and/or abnormal renal function are associated with worse outcomes in acute cardiac and trauma diseases [20,21]. Mid-term and long-term mortality models presented poorer performances as compared to short-term mortality models.

Models of prehospital and in-hospital mortality have explored age as an intrinsic hazard for adverse outcomes. Age seems to play an overlapping role in all patients in our three mortality groups. In agreement with previous studies, however, age is not a determining condition for mortality, whereas the burden of comorbidities prior to the event—a variable that has also been identified as a factor associated with morbidity in the short, mid, and long term—may be more critical [22,23]. In addition, other variables, such as elevated partial pressure of carbon dioxide and decreased prehospital hemoglobin, remained independent variables in all the analyzed time courses. Decreased hemoglobin has been shown to be a biomarker associated with enhanced frailty and pluripathology, increasing the risk of all-cause mortality in elderly patients, as well as a poor prognostic factor in acute pathologies [24]. Similarly, hypercapnia has been associated with tissue hypoperfusion in patients with respiratory diseases, both acute and chronic, showing increased mortality compared to patients with normocapnia [25]. On the other hand, the oxygen saturation/fraction of inspired oxygen ratio has already been validated as a predictive variable for early clinical deterioration in prehospital care [26], and the present study shows that this variable has a predictive capacity in the mid- and long-term, whereas oxygen saturation in isolation is only observed as a predictive variable in the short- and mid-term [27]. The models used in this work could a priori seem complex due to the number of variables included; however, the actual informatization of medicine could ease the inclusion of the models in daily practice. The integration of models into the monitor/device that captures all the information is now a reality that illustrates how our proposed models could help clinicians.

The study was not free of limitations. First, a convenience sample was made. To minimize bias, recruitment was performed in an uninterrupted way, involving several ambulance stations and hospitals (one minor general district hospital and three university tertiary hospitals) in urban and countryside areas, and involving cases with unselected acute disease. Despite this, the final result included a significantly aging population, a mirror representation of the population pyramid in Spain’s surrounding area [28]. Second, the data extractors were not blinded. To avoid possible cross-contamination, the EMS providers were unaware of the hospital follow-up data, and likewise, the hospital investigators were blinded to the variables collected during prehospital care. Only the data manager and PI had full access to the data. Third, the study was undertaken during the COVID-19 pandemic. SARS-CoV-2 has produced an exponential increase in pneumonia with multisystem involvement, ICU admissions, and excess mortality [29]. In addition, particularly during the first waves, the incidence of emergency calls to EMS dropped dramatically, so hidden mortality from acute life-threatening illness is plausible. Further studies are needed to quantify and identify the true pandemic dimension [30]. Finally, the models used are compounded by a large number of variables, a fact that is a handicap for clinical use in prehospital care. Additionally, to determine bedside analytical variables, the use of POCTs—which are devices of proven usefulness and reliability, but with a very uneven implementation—is mandatory, a circumstance that is capable of limiting the models’ utilization. To encourage the introduction of scoring systems or risk models (from simple clinical scores to complex models based on artificial intelligence), the future involves the incorporation in ambulances of portable computers that allow on-scene access to electronic medical records, with the option to use a variety of scores or models.

## 5. Conclusions

In summary, the developed models changed in terms of their components and predictive abilities with the outcome times. However, their performance allows us to state that prehospital variables provide excellent clinical and risk characterization at all the time points considered. This feedback may assist EMS providers in the complex process of on-scene decision-making, allowing them to provide personalized and customized care for each individual patient, starting from the initial steps of care.

## Figures and Tables

**Table 1:** Prehospital baseline predictors, mortality cluster vs. survivors.

		Noncumulative Mortality			
	Survivors	30-Day	180-Day	365-Day	*p* Value ^b^
No. (%) with data ^a^	3816 (79)	523 (10.8)	321 (6.6)	170 (3.6)	N.A.
Epidemiological variables					
Sex at birth, female	1615 (42.3)	200 (38.2)	127 (39.6)	60 (35.3)	0.088
Age, year	64 (48–78)	79 (67–86)	79 (68–87)	79 (69–86)	<0.001
Age groups, year					
18–49	1030 (27)	37 (7.1)	22 (6.9)	7 (4.1)	<0.001
50–74	1562 (40.9)	163 (31.2)	103 (32.1)	49 (28.8)	
>75	1224 (32.1)	323 (61.8)	196 (61.1)	114 (67.1)	
Zone, rural	1071 (28.1)	144 (27.5)	71 (22.1)	42 (24.7)	0.114
Transfer, ALS	2432 (6.5)	417 (79.7)	194 (60.4)	92 (54.1)	<0.001
Nursing homes	247 (6.5)	117 (22.4)	75 (23.4)	29 (17.1)	<0.001
aCCI, points	4 (1–6)	7 (5–9)	7 (5–9)	7 (5–10)	<0.001
On-scene vital signs					
RR, breaths/min	17 (14–21)	22 (15–29)	20 (16–28)	17 (14–24)	<0.001
SpO2, %	97 (95–98)	91 (81–96)	94 (89–96)	95 (92–97)	<0.001
SaFi	462 (448–467)	414 (319–452)	443 (382–457)	452 (429–462)	<0.001
SBP, mmHg	134 (126–152)	120 (90–149)	134 (110–152)	137 (113–158)	<0.001
DBP, mmHg	80 (67–90)	67 (53–87)	75 (60–90)	72 (61–87)	<0.001
MBP, mmHg	97 (85–110)	87 (66–108)	95 (78–109)	92 (86–109)	<0.001
Heart rate, beats/min	82 (70–100)	93 (74–120)	91 (75–110)	86 (70–110)	<0.001
Temperature, °C	36.1 (35.9–36.5)	36 (35.7–36.7)	36.2 (36–36.7)	36.3 (35.9–36.7)	<0.001
Glasgow coma scale, points					
Ocular	4 (4–4)	3 (1–4)	4 (3–4)	4 (4–4)	<0.001
Verbal	5 (5–5)	4 (1–5)	5 (5–5)	5 (5–5)	<0.001
Motor	6 (6–6)	6 (3–6)	6 (6–6)	6 (6–6)	<0.001
Baseline cardiac rhythm					
Sinus	2191 (57.4)	146 (27.9)	99 (30.8)	73 (42.9)	<0.001
Tachycardia ^c^	1306 (34.2)	319 (61)	193 (60.1)	78 (45.9)	
Bradycardia ^d^	267 (7)	48 (9.2)	19 (5.9)	12 (7.1)	
Pacemaker	52 (1.4)	10 (1.9)	10 (3.1)	7 (4.1)	
ST elevation	192 (5)	38 (7.3)	15 (4.7)	2 (1.2)	0.014
Prehospital blood test					
8	7.38 (7.34–7.42)	7.31 (7.12–7.38)	7.36 (7.32–7.42)	7.38 (7.33–7.42)	<0.001
pCO2, mmHg	39 (33–45)	46 (36–66)	43 (35–52)	41 (34–52)	<0.001
pO2, mmHg	33 (23–43)	23 (16–37)	28 (21–39)	29 (22–43)	<0.001
Bicarbonate, mEq	24.1 (22.1–26.8)	21.2 (16.9–25.3)	23.8 (21.1–27.7)	24.1 (21.6–26.6)	<0.001
Base excess (efc), mmol/L	0.6 (−1.9; 2)	−3.3 (−0.2; 0.8)	0.5 (−2.8; 2.6)	0.5 (−2.9; 2.1)	<0.001
Sodium, mmol/L	139 (137–140)	139 (135–141)	139 (136–140)	139 (135–140)	0.377
Potassium, mmol/L	4.1 (3.8–4.4)	4.2 (3.8–5)	4.1 (3.8–4.7)	4.2 (3.9–4.8)	<0.001
Calcium, mmol/L	1.15 (1.08–1.21)	1.11 (1.01–1.21)	1.13 (1.06–1.21)	1.15 (1.09–1.22)	<0.001
Chlorine, mmol/L	103 (100–105)	104 (100–108)	103 (100–106)	104 (100–106)	<0.001
TCO2, mmol/L	26 (23–28)	25 (20–31)	26 (23–31)	26 (23–30)	<0.001
Hemoglobin, g/dL	14.2 (13–15.7)	13.2 (11.4–14.8)	13.2 (11.5–14.8)	13.2 (12.1–14.8)	<0.001
Glucose, mg/dL	122 (104–151)	163 (130–227)	147 (118–206)	141 (112–177)	<0.001
Lactate, mmol/L	1.88 (1.17–2.98)	4.67 (3.03–7.72)	2.54 (1.87–3.54)	2.11 (1.42–2.98)	<0.001
Creatinine, mgr/dL	0.87 (0.76–1.11)	1.54 (1.07–2.37)	1.13 (0.86–1.54)	1.12 (0.81–1.54)	<0.001
Blood urea nitrogen, mg/dL	16 (12–21)	30 (20–41)	23 (16–33)	20 (13–29)	<0.001

Abbreviations: NA: not applicable; ALS: advanced life support; RR: respiratory rate; SPO2: oxygen saturation; SaFi: oxygen saturation/fraction of inspired oxygen ratio; SBP: systolic blood pressure; DBP: diastolic blood pressure; MBP: mean blood pressure; pCO2: partial pressure of carbon dioxide; pO2: partial pressure of oxygen; TCO2: total carbon dioxide content. ^a^ Values expressed as total numbers (percentage) or medians (25th–75th percentile), as appropriate. ^b^ The Mann‒Whitney U test or chi-squared test was used, as appropriate. ^c^ Tachycardia rhythm includes sinus tachycardia, atrial fibrillation, atrial flutter, supraventricular tachycardia, and ventricular tachycardia. ^d^ Bradycardia rhythm includes sinus bradycardia, first-degree atrioventricular (AV) block, Mobitz type I 2nd-degree AV block, Mobitz type II 2nd-degree AV block, and third-degree AV block.

**Table 2:** Numerical distribution of mortality according to age-adjusted Charlson comorbidity index.

		Noncumulative Mortality			
	Survivors	30-Day	180-Day	365-Day	*p* Value ^b^
No. (%) with data ^a^	3816 (79)	523 (10.8)	321 (6.6)	170 (3.6)	N.A.
aCCI (points)	4 (1–6)	7 (5–9)	7 (5–9)	7 (5–10)	<0.001
AIDS	39 (1)	7 (1.3)	1 (0.3)	4 (2.4)	0.181
Solid tumor, metastatic	67 (1.8)	46 (8.8)	52 (16.9)	16 (9.4)	<0.001
Liver disease, severe	112 (3.9)	38 (7.3)	15 (4.7)	11 (6.5)	<0.001
Lymphoma	31 (0.8)	8 (1.5)	11 (3.4)	6 (3.5)	<0.001
Leukemia	31 (0.8)	15 (2.9)	2 (0.6)	6 (3.5)	<0.001
Solid tumor, localized	525 (13.7)	121 (23.1)	90 (28)	43 (25.3)	<0.001
DM, end organ damage	306 (8)	96 (18.4)	52 (16.5)	32 (18.8)	<0.001
Severe CKD	311 (8.1)	101 (19.3)	53 (16.5)	36 (21.2)	<0.001
Hemiplegia	122 (3.2)	53 (10.1)	24 (7.5)	23 (13.5)	<0.001
DM, uncomplicated	481 (12.6)	90 (17.2)	54 (16.8)	29 (17.1)	0.003
Liver disease, mild	133 (2.5)	24 (4.6)	13 (4)	6 (3.5)	0.624
Peptic ulcer disease	314 (8.2)	73 (14)	27 (8.4)	25 (14.7)	<0.001
Connective disease	189 (5)	45 (8.6)	21 (6.5)	15 (8.8)	0.001
COPD	729 (19.1)	150 (28.7)	106 (33)	60 (35.3)	<0.001
Dementia	247 (6.5)	100 (19.7)	62 (19.3)	34 (20)	<0.001
Cerebrovascular disease	309 (8.1)	82 (15.7)	37 (11.5)	30 (17.6)	<0.001
Peripheral vascular disease	396 (10.4)	98 (14.9)	66 (20.6)	32 (18.8)	<0.001
Congestive heart failure	401 (10.4)	137 (26.2)	89 (27.7)	60 (35.3)	<0.001
Myocardial infarction	687 (18)	129 (24.7)	84 (26.2)	55 (32.4)	<0.001

Abbreviations: NA: not applicable; aCCI: age-adjusted Charlson comorbidity index; AIDS: acquired immunodeficiency syndrome; DM: diabetes mellitus; CKD: chronic kidney disease; COPD: chronic obstructive pulmonary disease. ^a^ Values expressed as total numbers (percentage) or medians (25th–75th percentile), as appropriate. ^b^ The Mann‒Whitney U test or chi-squared test was used, as appropriate.

**Table 3:** Principal outcomes and other determinants, mortality cluster vs. survivors.

		Noncumulative Mortality			
	Survivors	30-Day	180-Day	365-Day	*p* Value ^b^
No. (%) with data ^a^	3816 (79)	523 (10.8)	321 (6.6)	170 (3.6)	N.A.
Support on-scene					
NIMV	64 (1.7)	65 (12.4)	29 (9)	9 (5.3)	<0.001
IMV	115 (3)	160 (30.6)	22 (6.9)	5 (2.9)	<0.001
Vasoactive agents	30 (0.8)	85 (16.3)	6 (1.9)	2 (1.2)	<0.001
Suspected prehospital diagnose					
Abdominal pain/GB	139 (3.6)	17 (3.3)	15 (4.7)	9 (5.3)	<0.001
Abdominal trauma	17 (0.5)	4 (0.8)	1 (0.3)	1 (0.6)	
Acute chest pain	426 (11.2)	4 (0.8)	9 (2.8)	16 (9.4)	
Acute myocardial infarction	302 (7.9)	28 (5.4)	15 (4.7)	8 (4.7)	
Anaphylaxis	54 (1.4)	0 (0)	0 (0)	0 (0)	
Bradyarrhythmia	44 (1.2)	3 (0.6)	4 (1.2)	2 (1.2)	
Burns	17 (0.5)	4 (0.8)	0 (0)	1 (0.6)	
Cardiac arrest	14 (0.4)	54 (10.3)	7 (2.2)	0 (0)	
Congestive heart failure	21 (0.6)	23 (4.4)	10 (3.1)	5 (2.9)	
COPD/dyspnea	169 (4.4)	26 (5)	44 (13.7)	18 (10.6)	
Heart failure	132 (3.5)	34 (6.5)	29 (12.1)	15 (8.3)	
Hypertensive crisis	52 (1.4)	0 (0)	0 (0)	2 (1.2)	
Infection	72 (2)	12 (2.3)	14 (4.4)	12 (7.1)	
Metabolic disease	45 (1.2)	7 (1.3)	5 (1.6)	2 (1.2)	
Orthopedic trauma	261 (6.8)	2 (0.4)	10 (3.1)	3 (1.8)	
Poisoning	346 (9.1)	8 (1.5)	7 (2.2)	7 (4.1)	
Polytraumatized	86 (2.3)	24 (4.6)	3 (0.9)	1 (0.6)	
SARS-CoV-2	55 (1.4)	16 (3.1)	11 (3.4)	5 (2.9)	
Seizures	238 (6.2)	8 (1.5)	16 (5)	6 (3.5)	
Sepsis	78 (2)	102 (19.5)	29 (9)	10 (5.9)	
Status epilepticus	18 (0.5)	0 (0)	2 (0.6)	1 (0.6)	
Stroke	325 (8.5)	97 (18.5)	27 (8.4)	18 (10.6)	
Syncope	452 (11.8)	11 (2.1)	29 (9)	15 (8.8)	
Tachyarrhythmia	126 (3.3)	4 (0.8)	10 (3.1)	4 (2.4)	
Thoracic trauma	42 (1.1)	3 (0.6)	0 (0)	0 (0)	
Transient ischemic attack	98 (2.6)	3 (0.6)	6 (1.9)	4 (2.4)	
Trauma brain injury	182 (4.8)	29 (5.5)	8 (2.5)	5 (2.9)	
Hospital outcome					
Inpatient	1806 (47.3)	501 (95.8)	236 (73.5)	116 (68.2)	<0.001
ICU admission	304 (8)	189 (36.1)	41 (12.8)	8 (4.7)	<0.001
NIMV	65 (1.7)	57 (10.9)	30 (9.3)	10 (5.9)	<0.001
IMV	195 (5.1)	208 (39.8)	38 (11.8)	8 (4.7)	<0.001
Vasoactive agents	99 (2.6)	179 (34.2)	30 (9.3)	6 (3.5)	<0.001

Abbreviations: NA: not applicable; NIMV: noninvasive mechanical ventilation; IMV: invasive mechanical ventilation; GB: gastrointestinal bleeding; COPD: chronic obstructive pulmonary disease; SARS-CoV-2: severe acute respiratory syndrome coronavirus 2; ICU: intensive care unit. ^a^ Values expressed as total numbers (percentage). ^b^ Chi-squared test was used.

**Table 4:** Odds ratios of multivariate logistic regression.

30-Day	Odds Ratio	5% CI	95% CI	*p* Value
Age	1.043	1.035	1.052	<0.001
Respiratory rate	1.015	1.003	1.028	0.048
SaFi	0.992	0.990	0.994	<0.001
Heart rate	1.008	1.004	1.012	<0.001
Glasgow coma scale, Ocular	0.767	0.639	0.920	0.017
Glasgow coma scale, Verbal	0.750	0.666	0.846	<0.001
pCO2	1.016	1.008	1.023	<0.001
Calcium	0.117	0.053	0.258	<0.001
Chlorine	1.032	1.013	1.051	0.006
Hemoglobin	0.882	0.845	0.921	<0.001
Lactate	1.163	1.127	1.199	<0.001
Blood urea nitrogen	1.017	1.009	1.026	0.001
Creatinine	1.308	1.156	1.480	<0.001
aCCI	1.107	1.067	1.148	<0.001
180-day				
Age	1.018	1.009	1.026	0.001
Nursing homes	1.699	1.275	2.252	0.002
Respiratory rate	1.026	1.012	1.040	0.002
SpO2	0.967	0.955	0.980	<0.001
Glasgow coma scale, Verbal	0.857	0.782	0.942	0.007
Tachyarrhythmia	1.945	1.540	2.463	<0.001
pH	8.150	2.187	31.073	0.009
pCO2	1.012	1.004	1.021	0.019
Hemoglobin	0.870	0.832	0.909	<0.001
Lactate	1.093	1.047	1.139	0.001
Blood urea nitrogen	1.017	1.010	1.024	<0.001
aCCI	1.152	1.111	1.193	<0.001
365-day				
Age	1.021	1.010	1.032	0.002
SaFi	0.997	0.995	1.000	0.037
pCO2	1.014	1.003	1.024	0.027
Hemoglobin	0.904	0.854	0.958	0.004
aCCI	1.240	1.189	1.293	<0.001

Abbreviations: CI: confidence interval; aCCI: age-adjusted Charlson comorbidity index; pCO2: partial pressure of carbon dioxide; SaFi: oxygen saturation/fraction of inspired oxygen ratio; SpO2: oxygen saturation.

## Data Availability

Data can be obtained upon rationale request from the corresponding author.

## Supplementary Materials

The following supporting information can be downloaded at: https://www.mdpi.com/article/10.3390/diagnostics14121292/s1. Supplementary Figure S1, Study population flowchart; Supplementary Table S1, Summary descriptives table for 30-day mortality; Supplementary Table S2, Summary descriptives table for 180-day mortality; Supplementary Table S3, Summary descriptives table for 365-day mortality.
