# Supplementary material for: A Comparison of the Clinical Characteristics of Short-, Mid-, and Long-Term Mortality in Patients Attended by the Emergency Medical Services: An Observational Study
Source: Diagnostics (Basel). 2024 Jun 19;14(12):1292. doi: 10.3390/diagnostics14121292 (PMC11203341; doi:10.3390/diagnostics14121292)
Supplement: Supplementary file 1 [file diagnostics-14-01292-s001.zip › diagnostics-3027573-supplementary.pdf]

Supplementary data:

Table of contents:

Supplementary methods

Strengthening the Reporting of Observational Studies in Epidemiology (STROBE) Statement

Supplementary Results

- Supplementary Figure 1. Study population flowchart

- Supplementary table S1: Summary descriptives table for 30-day mortality

- Supplementary table S1: Summary descriptives table for 180-day mortality

- Supplementary table S1: Summary descriptives table for 365-day mortality

Supplementary Methods

STROBE Statement—checklist of items that should be included in reports of observational studies

	Item No	Recommendation
**Title and abstract**	1	(*a*) Indicate the study's design with a commonly used term in the title or the abstract
		(*b*) Provide in the abstract an informative and balanced summary of what was done and what was found
**Introduction**		
Background/rationale	2	Explain the scientific background and rationale for the investigation being reported
Objectives	3	State specific objectives, including any prespecified hypotheses
**Methods**		
Study design	4	Present key elements of study design early in the paper
Setting	5	Describe the setting, locations, and relevant dates, including periods of recruitment, exposure, follow-up, and data collection
Participants	6	(*a*) *Cohort study*—Give the eligibility criteria, and the sources and methods of selection of participants. Describe methods of follow-up<br>*Case-control study*—Give the eligibility criteria, and the sources and methods of case ascertainment and control selection. Give the rationale for the choice of cases and controls<br>*Cross-sectional study*—Give the eligibility criteria, and the sources and methods of selection of participants
		(*b*) *Cohort study*—For matched studies, give matching criteria and number of exposed and unexposed<br>*Case-control study*—For matched studies, give matching criteria and the number of controls per case
Variables	7	Clearly define all outcomes, exposures, predictors, potential confounders, and effect modifiers. Give diagnostic criteria, if applicable
Data sources/ measurement	8*	For each variable of interest, give sources of data and details of methods of assessment (measurement). Describe comparability of assessment methods if there is more than one group
Bias	9	Describe any efforts to address potential sources of bias

Study size	10	Explain how the study size was arrived at	
Quantitative variables	11	Explain how quantitative variables were handled in the analyses. If applicable, describe which groupings were chosen and why	
Statistical methods	12	(*a*) Describe all statistical methods, including those used to control for confounding	
		(*b*) Describe any methods used to examine subgroups and interactions	
		(*c*) Explain how missing data were addressed	
		(*d*) *Cohort study*—If applicable, explain how loss to follow-up was addressed<br>*Case-control study*—If applicable, explain how matching of cases and controls was addressed<br>*Cross-sectional study*—If applicable, describe analytical methods taking account of sampling strategy	
		(*e*) Describe any sensitivity analyses	

Continued on next page

**Results**			
Participants	13*	(a) Report numbers of individuals at each stage of study—eg numbers potentially eligible, examined for eligibility, confirmed eligible, included in the study, completing follow-up, and analysed	8
		(b) Give reasons for non-participation at each stage	8
		(c) Consider use of a flow diagram	8
Descriptive data	14*	(a) Give characteristics of study participants (eg demographic, clinical, social) and information on exposures and potential confounders	8
		(b) Indicate number of participants with missing data for each variable of interest	8
		(c) *Cohort study*—Summarise follow-up time (eg, average and total amount)	8
Outcome data	15*	*Cohort study*—Report numbers of outcome events or summary measures over time	*8*
		Case-control study—Report numbers in each exposure category, or summary measures of exposure	*8*
		Cross-sectional study—Report numbers of outcome events or summary measures	*8*
Main results	16	(*a*) Give unadjusted estimates and, if applicable, confounder-adjusted estimates and their precision (eg, 95% confidence interval). Make clear which confounders were adjusted for and why they were included	9
		(*b*) Report category boundaries when continuous variables were categorized	9
		(*c*) If relevant, consider translating estimates of relative risk into absolute risk for a meaningful time period	9
Other analyses	17	Report other analyses done—eg analyses of subgroups and interactions, and sensitivity analyses	9
**Discussion**			
Key results	18	Summarise key results with reference to study objectives	10-11
Limitations	19	Discuss limitations of the study, taking into account sources of potential bias or imprecision. Discuss both direction and magnitude of any potential bias	11-12
Interpretation	20	Give a cautious overall interpretation of results considering objectives, limitations, multiplicity of analyses, results from similar studies, and other relevant evidence	10,11,12
Generalisability	21	Discuss the generalisability (external validity) of the study results	11,12,13
**Other information**			
Funding	22	Give the source of funding and the role of the funders for the present study and, if applicable, for the original study on which the present article is based	2

*Give information separately for cases and controls in case-control studies and, if applicable, for exposed and unexposed groups in cohort and cross-sectional studies.

Note: An Explanation and Elaboration article discusses each checklist item and gives methodological background and published examples of transparent reporting. The STROBE checklist is best used in conjunction with this article (freely available on the Web sites of PLoS Medicine at http://www.plosmedicine.org/, Annals of Internal Medicine at

http://www.annals.org/, and Epidemiology at http://www.epidem.com/). Information on the STROBE Initiative is available at www.strobe-statement.org.

Figure S1. Flowchart of the participants in the study

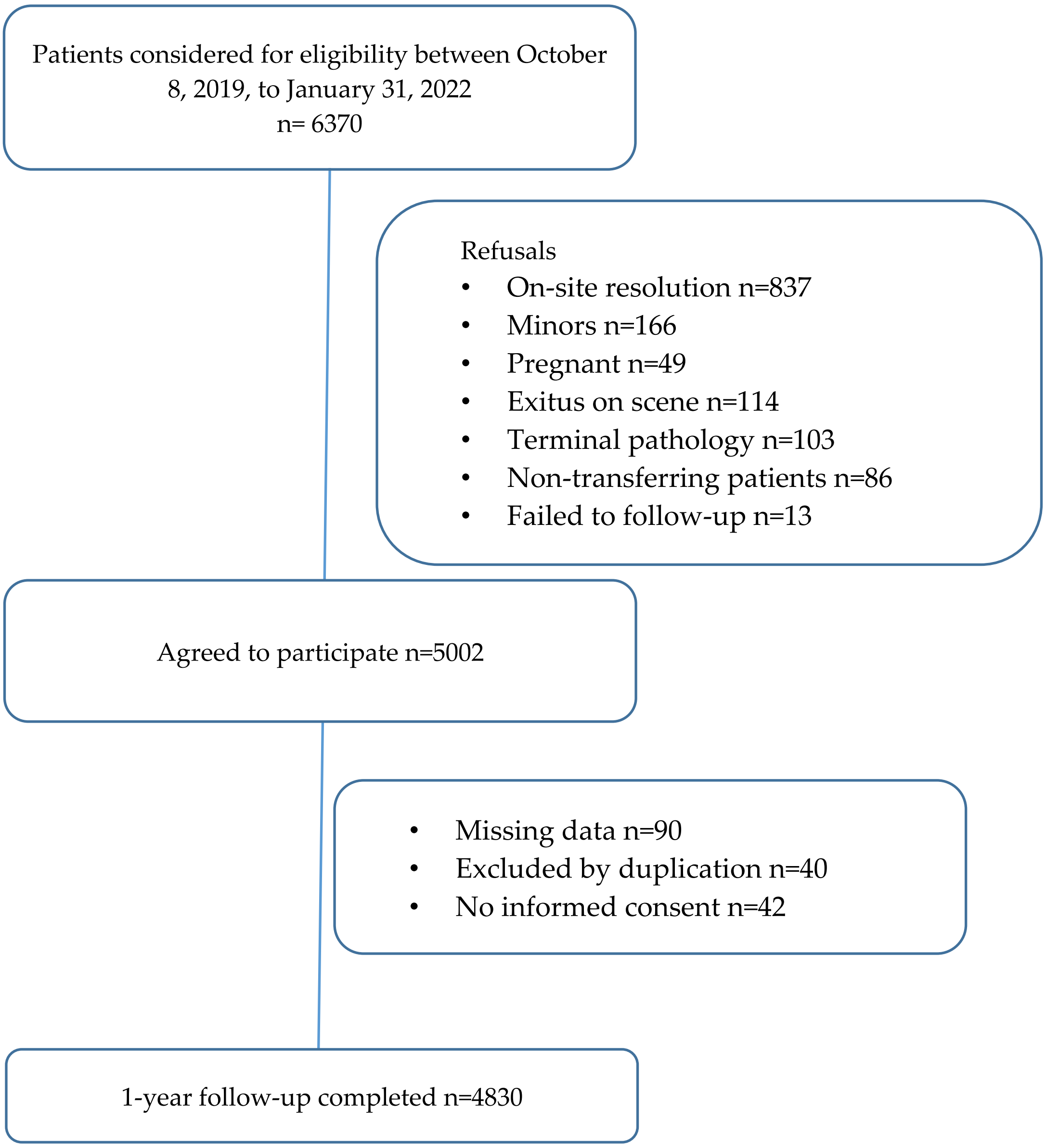

Supplementary Table S1: Summary descriptives table for 30-day mortality

	**Survivors** [a] *N=3813*	**Non-survivors** [a] *N=523*	**pvalue**[b]
Age	61.5 (19.9)	75.1 (15.3)	<0.001
Sex:			0.088
Male	2201 (57.7%)	323 (61.8%)	
Female	1612 (42.3%)	200 (38.2%)	
Zone:			0.831
Non-rural	2742 (71.9%)	379 (72.5%)	
Rural	1071 (28.1%)	144 (27.5%)	
Nursing homes:			<0.001
No	3566 (93.5%)	406 (77.6%)	
Yes	247 (6.48%)	117 (22.4%)	
RR, breaths/min	18.6 (6.73)	22.5 (10.7)	<0.001
SpO2, %	95.5 (5.69)	86.1 (13.4)	<0.001
SaFi	0.22 (0.05)	0.27 (0.16)	<0.001
SBP, mmHg	448 (47.4)	371 (105)	<0.001
DBP, mmHg	97.8 (20.5)	86.9 (31.3)	<0.001
MBP, mmHg	86.9 (26.9)	97.7 (38.4)	<0.001
Heart rate, beats/min	36.2 (0.79)	36.2 (1.17)	0.516
Glasgow coma scale Ocular	3.77 (0.64)	2.80 (1.27)	<0.001
Glasgow coma scale Verbal	4.66 (0.96)	3.25 (1.75)	<0.001
Glasgow coma scale Motor	5.78 (0.83)	4.43 (1.95)	<0.001
Baseline cardiac rhythm			<0.001
Sinus	2190 (57.4%)	146 (27.9%)	
Tachycardia[c]	1304 (34.2%)	319 (61.0%)	
Bradycardia[d]	267 (7.00%)	48 (9.18%)	

	Survivors [a] N=3813	Non-survivors [a] N=523	pvalue[b]
Pacemaker	52 (1.36%)	10 (1.91%)	
ST elevation:			0.042
No	3621 (95.0%)	485 (92.7%)	
Yes	192 (5.04%)	38 (7.27%)	
pH	7.37 (0.09)	7.24 (0.20)	<0.001
pCO2, mmHg	40.3 (11.4)	52.6 (20.7)	<0.001
pO2, mmHg	35.2 (14.3)	29.2 (17.0)	<0.001
Bicarbonate, mEq	24.3 (4.02)	21.3 (6.34)	<0.001
Base excess (efc), mmol/L	-0.26 (4.12)	-4.47 (7.89)	<0.001
Sodium, mmol/L	139 (3.64)	139 (6.85)	0.853
Potassium, mmol/L	4.17 (0.65)	4.50 (1.19)	<0.001
Calcium, mmol/L	1.14 (0.10)	1.10 (0.17)	<0.001
Chlorine, mmol/L	103 (4.04)	104 (7.33)	<0.001
TCO2, mmol/L	25.9 (4.78)	26.2 (8.10)	0.381
Hemoglobin, g/dL	14.2 (2.11)	13.0 (2.83)	<0.001
Glucose, mg/dL	138 (64.7)	189 (97.8)	<0.001
Lactate, mmol/L	2.64 (2.60)	6.07 (4.40)	<0.001
Blood urea nitrogen mg/dL	18.5 (10.9)	33.9 (18.6)	<0.001
Creatinine, mgr/dL	1.02 (0.60)	1.93 (1.46)	<0.001
aCCI	3.90 (3.28)	7.05 (3.62)	<0.001

Abbreviations: NA: not applicable; ALS: advanced life support; aCCI: age Age-Charlson comorbidity index; RR: respiratory rate; SPO2: oxygen saturation; SaFi: oxygen saturation/fraction of inspired oxygen *ratio*; SBP: systolic blood pressure; DBP: diastolic blood pressure; MBP: mean blood pressure; pCO2: partial pressure of carbon dioxide; pO2: partial pressure of oxygen; TCO2: total carbon dioxide content.

[a]Values expressed as total number (percentage) and mean (standard deviation), as appropriate.

[b]The Mann–Whitney U test or chi-squared test was used as appropriate.

[c]Tachycardia rhythm includes sinus tachycardia, atrial fibrillation, atrial flutter, supraventricular tachycardia, and ventricular tachycardia.

[d]Bradycardia rhythm includes sinus bradycardia, first-degree atrioventricular (AV) block, Mobitz type I 2nd-degree AV block, Mobitz type II 2nd-degree AV block, and third-degree AV block.

Supplementary Table S2: Summary descriptives table for 180-day mortality

	**Survivors** [a] *N=3813*	**Non-survivors** [a] *N=321*	**pvalue**[b]
Age	61.5 (19.9)	75.7 (14.3)	<0.001
Sex:			0.375
Male	2201 (57.7%)	194 (60.4%)	
Female	1612 (42.3%)	127 (39.6%)	
Zone:			0.026
Non-rural	2742 (71.9%)	250 (77.9%)	
Rural	1071 (28.1%)	71 (22.1%)	
Nursing homes:			<0.001
No	3566 (93.5%)	246 (76.6%)	
Yes	247 (6.48%)	75 (23.4%)	
RR, breaths/min	18.6 (6.73)	22.6 (9.16)	<0.001
SpO2, %	95.5 (5.69)	90.4 (9.92)	<0.001
SaFi	0.22 (0.05)	0.23 (0.08)	0.014
SBP, mmHg	448 (47.4)	412 (72.2)	<0.001
DBP, mmHg	97.8 (20.5)	94.0 (23.3)	0.004
MBP, mmHg	86.9 (26.9)	94.8 (30.0)	<0.001
Heart rate, beats/min	36.2 (0.79)	36.3 (0.98)	0.007
Glasgow coma scale Ocular	3.77 (0.64)	3.55 (0.86)	<0.001
Glasgow coma scale Verbal	4.66 (0.96)	4.33 (1.33)	<0.001
Glasgow coma scale Motor	5.78 (0.83)	5.53 (1.25)	<0.001
Baseline cardiac rhythm			<0.001

	Survivors [a] N=3813	Non-survivors [a] N=321	pvalue[b]
Sinus	2190 (57.4%)	99 (30.8%)	
Tachycardia[c]	1304 (34.2%)	193 (60.1%)	
Bradycardia[d]	267 (7.00%)	19 (5.92%)	
Pacemaker	52 (1.36%)	10 (3.12%)	
ST elevation:			0.879
No	3621 (95.0%)	306 (95.3%)	
Yes	192 (5.04%)	15 (4.67%)	
pH	7.37 (0.09)	7.35 (0.12)	<0.001
pCO2, mmHg	40.3 (11.4)	46.6 (16.4)	<0.001
pO2, mmHg	35.2 (14.3)	31.1 (14.7)	<0.001
Bicarbonate, mEq	24.3 (4.02)	24.2 (5.38)	0.940
Base excess (efc), mmol/L	-0.26 (4.12)	-0.41 (5.59)	0.633
Sodium, mmol/L	139 (3.64)	139 (5.04)	0.820
Potassium, mmol/L	4.17 (0.65)	4.26 (0.81)	0.039
Calcium, mmol/L	1.14 (0.10)	1.13 (0.13)	0.033
Chlorine, mmol/L	103 (4.04)	103 (5.12)	0.043
TCO2, mmol/L	25.9 (4.78)	27.4 (6.22)	<0.001
Hemoglobin, g/dL	14.2 (2.11)	13.1 (2.68)	<0.001
Glucose, mg/dL	138 (64.7)	173 (92.4)	<0.001
Lactate, mmol/L	2.64 (2.60)	3.39 (2.94)	<0.001
Blood urea nitrogen mg/dL	18.5 (10.9)	27.3 (16.6)	<0.001
Creatinine, mgr/dL	1.02 (0.60)	1.38 (0.99)	<0.001
aCCI	3.90 (3.28)	7.28 (3.42)	<0.001

Abbreviations: NA: not applicable; ALS: advanced life support; aCCI: age Age-Charlson comorbidity index; RR: respiratory rate; SPO2: oxygen saturation; SaFi: oxygen saturation/fraction of inspired oxygen *ratio*; SBP: systolic blood pressure; DBP: diastolic blood pressure; MBP: mean blood pressure; pCO2: partial pressure of carbon dioxide; pO2: partial pressure of oxygen; TCO2: total carbon dioxide content.

[a]Values expressed as total number (percentage) and mean (standard deviation), as appropriate.
[b]The Mann–Whitney U test or chi-squared test was used as appropriate.
[c]Tachycardia rhythm includes sinus tachycardia, atrial fibrillation, atrial flutter, supraventricular tachycardia, and ventricular tachycardia.
[d]Bradycardia rhythm includes sinus bradycardia, first-degree atrioventricular (AV) block, Mobitz type I 2nd-degree AV block, Mobitz type II 2nd-degree AV block, and third-degree AV block.

Supplementary Table S3: Summary descriptives table for 365-day mortality

	**Survivors** [a] *N=3813*	**Non-survivors** [a] *N=170*	**pvalue**[b]
Age	61.5 (19.9)	76.2 (13.6)	<0.001
Sex:			0.084
Male	2201 (57.7%)	110 (64.7%)	
Female	1612 (42.3%)	60 (35.3%)	
Zone:			0.382
Non-rural	2742 (71.9%)	128 (75.3%)	
Rural	1071 (28.1%)	42 (24.7%)	
Nursing homes:			<0.001
No	3566 (93.5%)	141 (82.9%)	
Yes	247 (6.48%)	29 (17.1%)	
RR, breaths/min	18.6 (6.73)	19.7 (7.71)	0.071
SpO2, %	95.5 (5.69)	93.3 (6.66)	<0.001
SaFi	0.22 (0.05)	0.23 (0.09)	0.068
SBP, mmHg	448 (47.4)	426 (69.0)	<0.001
DBP, mmHg	97.8 (20.5)	95.6 (21.5)	0.188
MBP, mmHg	86.9 (26.9)	89.0 (25.4)	0.291
Heart rate, beats/min	36.2 (0.79)	36.4 (1.03)	0.003
Glasgow coma scale Ocular	3.77 (0.64)	3.66 (0.78)	0.072
Glasgow coma scale Verbal	4.66 (0.96)	4.42 (1.25)	0.013

	Survivors [a]	Non-survivors [a]	pvalue[b]
	N=3813	*N=170*	
Glasgow coma scale Motor	5.78 (0.83)	5.67 (1.00)	0.153
Baseline cardiac rhythm			<0.001
Sinus	2190 (57.4%)	73 (42.9%)	
Tachycardia[c]	1304 (34.2%)	78 (45.9%)	
Bradycardia[d]	267 (7.00%)	12 (7.06%)	
Pacemaker	52 (1.36%)	7 (4.12%)	
ST elevation:			0.035
No	3621 (95.0%)	168 (98.8%)	
Yes	192 (5.04%)	2 (1.18%)	
pH	7.37 (0.09)	7.36 (0.10)	0.146
pCO2, mmHg	40.3 (11.4)	45.2 (15.4)	<0.001
pO2, mmHg	35.2 (14.3)	34.0 (15.6)	0.328
Bicarbonate, mEq	24.3 (4.02)	24.2 (4.57)	0.936
Base excess (efc), mmol/L	-0.26 (4.12)	-0.36 (4.72)	0.787
Sodium, mmol/L	139 (3.64)	138 (4.46)	0.106
Potassium, mmol/L	4.17 (0.65)	4.33 (0.75)	0.007
Calcium, mmol/L	1.14 (0.10)	1.15 (0.09)	0.278
Chlorine, mmol/L	103 (4.04)	103 (4.78)	0.625
TCO2, mmol/L	25.9 (4.78)	27.3 (5.62)	0.002
Hemoglobin, g/dL	14.2 (2.11)	13.3 (2.43)	<0.001
Glucose, mg/dL	138 (64.7)	163 (83.7)	<0.001
Lactate, mmol/L	2.64 (2.60)	2.85 (2.75)	0.319
Blood urea nitrogen mg/dL	18.5 (10.9)	23.5 (12.7)	<0.001
Creatinine, mgr/dL	1.02 (0.60)	1.31 (0.76)	<0.001
aCCI	3.90 (3.28)	7.69 (3.21)	<0.001

Abbreviations: NA: not applicable; ALS: advanced life support; aCCI: age Age-Charlson comorbidity index; RR: respiratory rate; SPO2: oxygen saturation; SaFi: oxygen saturation/fraction of inspired oxygen *ratio*; SBP: systolic blood pressure; DBP: diastolic blood pressure; MBP: mean blood pressure; pCO2: partial pressure of carbon dioxide; pO2: partial pressure of oxygen; TCO2: total carbon dioxide content.

[a]Values expressed as total number (percentage) and mean (standard deviation), as appropriate.

[b]The Mann–Whitney U test or chi-squared test was used as appropriate.

[c]Tachycardia rhythm includes sinus tachycardia, atrial fibrillation, atrial flutter, supraventricular tachycardia, and ventricular tachycardia.

[d]Bradycardia rhythm includes sinus bradycardia, first-degree atrioventricular (AV) block, Mobitz type I 2nd-degree AV block, Mobitz type II 2nd-degree AV block, and third-degree AV block.
